# Ventricular flow analysis and its association with exertional capacity in repaired tetralogy of Fallot: 4D flow cardiovascular magnetic resonance study

**DOI:** 10.1186/s12968-021-00832-2

**Published:** 2022-01-03

**Authors:** Xiaodan Zhao, Liwei Hu, Shuang Leng, Ru-San Tan, Ping Chai, Jennifer Ann Bryant, Lynette L. S. Teo, Marielle V. Fortier, Tee Joo Yeo, Rong Zhen Ouyang, John C. Allen, Marina Hughes, Pankaj Garg, Shuo Zhang, Rob J. van der Geest, James W. Yip, Teng Hong Tan, Ju Le Tan, Yumin Zhong, Liang Zhong

**Affiliations:** 1 National Heart Research Institute Singapore, National Heart Centre Singapore, Singapore, Singapore; 2 Department of Radiology, Shanghai Children’s Medical Center, School of Medicine, Shanghai Jiao Tong University, Shanghai, China; 3 Duke-NUS Medical School, Singapore, Singapore; 4 National University Hospital Singapore, Singapore, Singapore; 5 KK Women’s and Children’s Hospital, Singapore, Singapore; 6 Singapore Institute for Clinical Sciences, A*STAR, Singapore, Singapore; 7 Department of Cardiovascular Medicine, University of East Anglia, Norwich, UK; 8 Philips Healthcare Germany, Hamburg, Germany; 9 Department of Radiology, Leiden University Medical Center, Leiden, Netherlands

**Keywords:** Repaired tetralogy of Fallot, Kinetic energy, 4D flow CMR, Flow components, Cardiopulmonary exercise testing

## Abstract

**Background:**

Four-dimensional (4D) flow cardiovascular magnetic resonance (CMR) allows quantification of biventricular blood flow by flow components and kinetic energy (KE) analyses. However, it remains unclear whether 4D flow parameters can predict cardiopulmonary exercise testing (CPET) as a clinical outcome in repaired tetralogy of Fallot (rTOF). Current study aimed to (1) compare 4D flow CMR parameters in rTOF with age- and gender-matched healthy controls, (2) investigate associations of 4D flow parameters with functional and volumetric right ventricular (RV) remodelling markers, and CPET outcome.

**Methods:**

Sixty-three rTOF patients (14 paediatric, 49 adult; 30 ± 15 years; 29 M) and 63 age- and gender-matched healthy controls (14 paediatric, 49 adult; 31 ± 15 years) were prospectively recruited at four centers. All underwent cine and 4D flow CMR, and all adults performed standardized CPET same day or within one week of CMR. RV remodelling index was calculated as the ratio of RV to left ventricular (LV) end-diastolic volumes. Four flow components were analyzed: direct flow, retained inflow, delayed ejection flow and residual volume. Additionally, three phasic KE parameters normalized to end-diastolic volume (KEi_EDV_), were analyzed for both LV and RV: peak systolic, average systolic and peak E-wave.

**Results:**

In comparisons of rTOF vs. healthy controls, median LV retained inflow (18% vs. 16%, *P* = 0.005) and median peak E-wave KEi_EDV_ (34.9 µJ/ml vs. 29.2 µJ/ml, *P* = 0.006) were higher in rTOF; median RV direct flow was lower in rTOF (25% vs. 35%, *P* < 0.001); median RV delayed ejection flow (21% vs. 17%, *P* < 0.001) and residual volume (39% vs. 31%, *P* < 0.001) were both greater in rTOF. RV KEi_EDV_ parameters were all higher in rTOF than healthy controls (all *P* < 0.001). On multivariate analysis, RV direct flow was an independent predictor of RV function and CPET outcome. RV direct flow and RV peak E-wave KEi_EDV_ were independent predictors of RV remodelling index.

**Conclusions:**

In this multi-scanner multicenter 4D flow CMR study, reduced RV direct flow was independently associated with RV dysfunction, remodelling and, to a lesser extent, exercise intolerance in rTOF patients. This supports its utility as an imaging parameter for monitoring disease progression and therapeutic response in rTOF.

*Clinical Trial Registration* https://www.clinicaltrials.gov. Unique identifier: NCT03217240.

**Supplementary Information:**

The online version contains supplementary material available at 10.1186/s12968-021-00832-2.

## Introduction

Tetralogy of Fallot (TOF) is the most common form of cyanotic congenital heart disease (CHD) accounting for approximately one in every 3600 livebirths and 5–7% of all CHD. It affects males and females equally [1, 2]. With advances in diagnosis and postoperative care, those born with TOF can now undergo corrective surgical repair early in life and expect to survive to adulthood. Surgical correction of TOF typically involves ventricular septal defect closure, resection of obstructive right ventricular (RV) outflow tract musculature, and pulmonary valvotomy or placement of a transannular patch.

Pulmonary regurgitation (PR) is a common complication in repaired TOF (rTOF), especially with transannular patch repair, that may occur many years after the primary repair procedure. Significant postoperative PR has been found to be associated with progressive RV dilation and dysfunction, as well as risk of sustained ventricular tachycardia and sudden death [3]. Adverse remodelling initially involves RV but eventually affects the left ventricle (LV) as well, as both ventricles share a common septum and are encased within the pericardial cavity. Ventricular-ventricular interaction results in alterations of diastolic [4] and systolic function [5], and LV hemodynamics. Cardiovascular magnetic resonance (CMR) possesses excellent reproducibility for ventricular volume measurement and is the reference standard for quantification of ventricular size and function in rTOF patients [6]. Although pulmonary homograft replacement at guideline-recommended RV volume threshold values may reverse RV remodelling, the evidence for mortality benefit is less firm [7]. Cardiopulmonary exercise testing (CPET) offers additional prognostic guidance for timing of intervention [8] but is not routinely performed in the asymptomatic or mildly symptomatic patient. In a retrospective analysis for rTOF patients with both CMR and CPET, RV ejection fraction (RVEF) < 40% but neither RV end-diastolic volume (EDV) nor end-systolic volume (ESV) was predictive of peak oxygen uptake (VO_2_) below the established prognostic threshold of 27 ml/kg/min [9]. As severe RV dysfunction presents late, its diagnostic utility for incipient exercise intolerance is limited. Factors other than PR may underpin functional deterioration in rTOF patients. In a study of 81 paediatric rTOF patients, electrocardiographic (ECG) right bundle branch block-induced prolonged QRS duration, more than PR, was associated with reductions in RVEF and peak VO_2_ [10]. In these studies, intracardiac flow features were not investigated in rTOF patients.

Four-dimensional (4D) flow CMR quantifies blood flow in three orthogonal directions simultaneously within a volume of interest in a time-resolved manner throughout the cardiac cycle. It allows comprehensive visualization of multidirectional flow patterns within the heart and reads out the velocities of multiple jets in 3D space without the need to align the velocity-encoding direction, and may thus yield higher diagnostic accuracy than standard 2D flow CMR or Doppler echo for quantification of pulmonary artery pressure in patients with pulmonary hypertension [11]. 4D flow CMR patterns can be characterized in terms of flow velocities and volume, flow components and kinetic energy (KE) loss. Compared with LV direct flow, RV direct flow contributed to a larger portion of the RV EDV, and is the main determinant of RV intracavity blood flow KE [12]. Altered blood flow and flow velocities as well as pathological vortices were observed in ten rTOF patients compared with four healthy subjects [13]. Other studies of rTOF patients reported derangements of LV and RV KE [14, 15], abnormal LV diastolic direct flow and increased recirculating residual volume in mild to moderate RV dilation [16]. In a recent study of 58 rTOF [17], the RV was systematically segmented into RV outflow (RVOT) and inflow components, and the respective 4D flow data were analyzed that could quantify intracardiac vorticity and energy loss [18]. Diastolic to systolic quotients of RVOT vorticities and viscous energy losses, both attributable to PR, were found to be significant negatively correlated with peak VO_2_ and % predicted peak VO_2_ [17].

We aimed to compare 4D flow measurements in rTOF patients versus age- and gender-matched healthy controls, and investigate the association of these measurements with standard volume and function indexes of RV remodelling as well as quantitative outcomes of CPET.

## Methods

### Study population

**Fig. 1:**
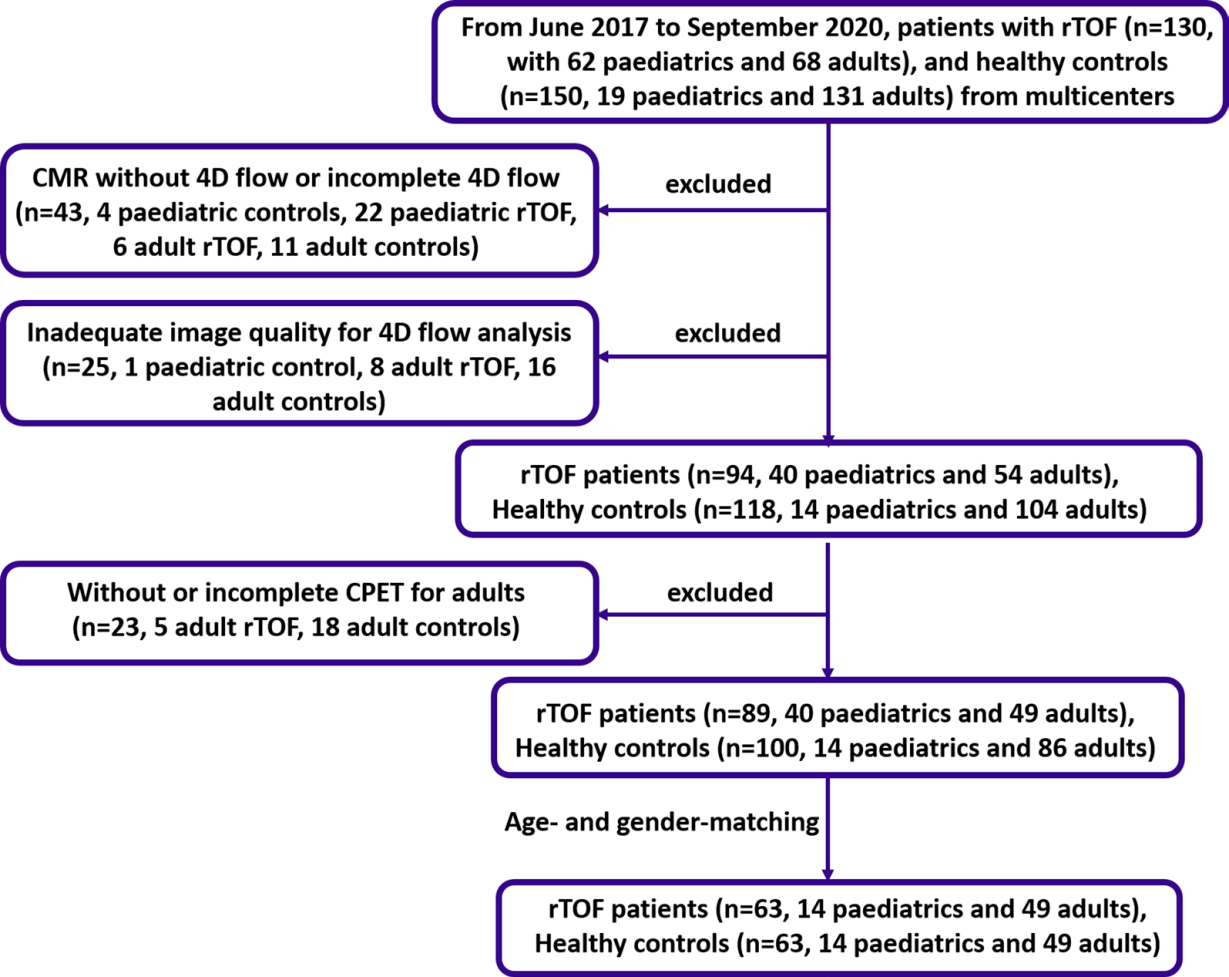
Flow chart. *4D* four-dimensional, *CMR* cardiovascular magnetic resonance, *CPET* cardiopulmonary exercise testing, *rTOF* repaired tetralogy of Fallot

From June 2017 to September 2020, 130 patients with rTOF and 150 healthy subjects were prospectively recruited from four centers. After applying exclusion criteria (Fig. 1), 63 rTOF patients (14 paediatric; 49 adult) and 63 age- and gender-matched healthy controls (14 paediatric; 49 adult) were selected to be included in the current analysis. The healthy control group all had no prior cardiovascular disease, pulmonary disease, hypertension, or diabetes mellitus. Additionally, all adult rTOF patients and healthy controls underwent CPET. Detailed inclusion and exclusion criteria for healthy subjects and rTOF patients had been previously registered at ClinicalTrials.gov (Identifier: NCT03217240). The study protocol had been approved by site Institutional Review Boards. Written informed consent was obtained from all subjects or, where applicable, their parents or legal guardians.

### Cardiovascular ac magnetic resonance protocol

CMR acquisition was performed with agreed, standardized protocols on all major vendor scanners from the different sites using a 3 T (Ingenia, Philips Healthcare, Best, the Netherlands), 1.5 T (Magneton Aera, Siemens Healthineers, Erlangen, Germany), or 3 T (Discovery, General Electric Healthcare, Chicago, Illinois, USA) CMR scanner, respectively. 2D balanced steady state free precession end-expiratory breath-held short-axis cine images covering the LV and RV from base to apex were acquired along with 2-, 3- and 4-chamber long-axis and RVOT cine images with a temporal resolution of 30 frames per cardiac cycle. Standard through-plane 2D  phase contrast (PC) flow measurement was acquired just above the pulmonary valve in a plane perpendicular to the long axis of the pulmonary trunk. Whole-heart 4D flow CMR was performed with free breathing without respiratory navigator gating following guideline recommendations [19, 20]. Typical 4D flow acquisition parameters are detailed in Table 1.

**Table 1:** 4D flow CMR imaging parameters in this study

Vendor	Philips	Siemens	GE
Magnetic field strength (T)	3	1.5	3
Pulse sequence	Spoiled gradient echo	Spoiled gradient echo	Spoiled gradient echo
Acceleration method	EPI factor 5 and SENSE factor 2	GRAPPA factor 2	kat-ARC factor 2
Field of view (mm^2^)	340 × 340	340 × 236.8	340 × 340
Slice orientation	Coronal	Sagittal	Axial
Acquired voxel size (mm^3^)	3.0 × 3.0 × 3.0	3.0 × 3.0 × 3.0	1.4–2.0 × 1.4–2.0 × 1.4–2.0
TR/TE (ms)	6.4–12.0/3.3–4.0	40.56/2.94	4.3/2.1
Flip angle (°)	10	9	8–12
Cardiac phases	30	27–33 (depending on RR interval)	30
VENC (cm/s)	150 (maximum 220)	220	160–200
Cardiac gating	Retrospective ECG gating	Prospective ECG gating	Retrospective ECG gating
Respiratory motion	Free breathing	Free breathing	Free breathing
Scan time (min)	5—10	20	6–10

*CMR* cardiovascular magnetic resonance, *ECG* electrocardiogram, *EPI* echo-planar imaging, *GRAPPA* generalized autocalibrating partially parallel acquisition, *kat-ARC* k-adaptive-t autocalibrating reconstruction for Cartesian sampling, *SENSE* sensitivity encoding, *TE* echo time, *TR* repetition time, *VENC* velocity encoding

### CMR data analysis

#### Biventricular volume analysis

Expert analysis of the imaging data from all sites was performed within one core laboratory. LV and RV volumes and systolic function were measured offline using MASS (version 2019EXP, Leiden University Medical Center, Leiden, The Netherlands) using the protocol previously published [21]. Papillary and trabecular muscles were included as LV and RV volume. RV remodelling index was determined as the ratio of RVEDV to LVEDV [22]. Cut-off values of RVEDV/LVEDV ratio for moderate-to-severe and severe RV remodelling were 1.41 and 2.0, respectively [22]. Tricuspid regurgitation (TR) was visually graded as none, mild, moderate, or severe [23] and the presence of RVOT dyskinesia was assessed by experts (RST, YMZ). Pulmonary valve annulus (PVA) diameter was measured in the RVOT view at end diastole [24]. The inter-ventricular mechanical synchrony index was measured as the time difference to maximal displacement between RV free wall and LV lateral wall using feature tracking in four-chamber long-axis cine images [25–27].

#### 2D and 4D flow analyses

Standard 2D PC flow and 4D flow images were analyzed using MASS. 2D PC flow images generated main pulmonary artery flow curve, from which PR volume (PRV) and regurgitant fraction (PRF) were extracted. RV restrictive physiology was identified by the presence of end-diastolic forward flow by experts (RST, YMZ). Main pulmonary artery pulse wave velocity (PWV) was calculated from the slope of the line fitted to the flow-area data, which represents the ratio of flow to area changes during early systole [28].

Error corrections and 4D flow quality checks were performed as previously reported [29–31]. Phasic endocardial and epicardial contours from LV and RV volume curves were used for flow component and KE analyses. The positions of path lines at end systole were used to classify flow into 4 functional components [32, 33]: (1) direct flow: blood that enters and exits the ventricle in the analyzed cardiac cycle; (2) retained inflow: enters the ventricle but does not exit during the analyzed cycle; (3) delayed ejection flow: starts within the ventricle and exits during the analyzed cycle; and (4) residual volume: blood that remained in the ventricle for the duration of at least one full cardiac cycle. Each component volume was indexed as a proportion of the total ventricular EDV. Movies with appropriate color legend for RV four flow components in one normal subject and one rTOF patient were provided in Additional file 1.

For each volumetric element (voxel), KE was computed using the following formula:

$$\mathrm{KE}=\frac{1}{2}{\uprho }_{\mathrm{blood}}\cdot {\mathrm{V}}_{\mathrm{voxel}}\cdot {v}_{\mathrm{voxel}}^{2},$$

where ${\uprho }_{\mathrm{blood}}$ is the blood density (1.06 g/cm^3^); ${\mathrm{V}}_{\mathrm{voxel}}$ voxel volume; and ${v}_{\mathrm{voxel}}$ velocity of the corresponding voxel. All KE parameters are normalized to EDV (KEi_EDV_) and presented in μJ/ml. KEi_EDV_ parameters—peak systole, average systole and peak E-wave—were extracted from time-resolved KE curves. We further define KE discordance as the RV/LV systolic KEi_EDV_ ratio. Inter-ventricular flow synchrony assessment is based on the difference between RV and LV in time to minimal KE during systole normalized to EDV**.**

### Cardiopulmonary exercise testing

All adult subjects underwent exercise testing to maximal volition, on a Lode BV Corival electronically braked cycle ergometer (Gronigen, Netherlands) within one week of the CMR scan. A ramp protocol was adapted for each subject as previously described [34]. Minute ventilation (VE), VO_2_, and carbon dioxide output (VCO_2_) were acquired breath-by-breath, and averaged over 10-s intervals. Predicted peak VO_2_ was calculated based on proposed normative values [35, 36]. VE/VCO_2_ slope was calculated via least squares linear regression (y = mx + b, m = slope) using VE and VCO_2_ values acquired from the initiation of exercise to peak. Patients were stratified by peak VO_2_ values into two groups: low risk: peak VO_2_ > 15 ml/kg/min; medium to high risk: peak VO_2_ ≤ 15 ml/kg/min [37]. The exercise capacity with intermediate and high risks was determined as % predicted peak VO_2_ ≤ 65% [38].

### Reproducibility

20 subjects were randomly selected to conduct the reproducibility analysis using the coefficient of variation (CV), which was calculated as the overall residual standard deviation divided by the overall mean of the observations. To assess inter-observer variability, LV and RV endocardial and epicardial contours were segmented by a second independent observer (SL) blinded to the first observer’s results. A second segmentation was performed by the primary observer (XDZ) one month after the initial segmentation to assess intra-observer variability.

### Statistical analysis

Data were analyzed using SPSS (version 22.0, Statistical Package for the Social Sciences, International Business Machines, Inc., Armonk, New York, USA). Continuous variables were summarized as mean ± standard deviation (SD) or median (interquartile range), as appropriate. For normally distributed data, the two-sample t-test was used to compare means between two independent groups. For non-normally distributed data, the Mann–Whitney U-Test was used for comparisons between two groups and the Kruskal–Wallis (K-W) non-parametric one-way ANOVA for more than two groups (RVEF subgroups vs healthy controls, RVEDV/LVEDV ratio subgroups vs healthy controls, peak VO_2_ subgroups vs healthy controls) with post-hoc pair-wise comparisons in the event of a significant K-W test. The chi-square test or Fisher’s exact test, as appropriate, were used for analysis of categorical variables. Associations between continuous variables were assessed using Pearson correlation analysis. Univariate and stepwise multivariable linear regression analyses were used to identify independent predictors of RV dysfunction, RV remodelling index and exercise capacity. Regression residuals were assessed visually for variance homogeneity and via Q–Q plots for normality. Assumptions of variance homogeneity and approximate normality of residuals were found to be tenable overall with adequate sample size to confer normality on coefficient estimates, ensuring valid hypothesis tests and confidence intervals. Receiver operator characteristic (ROC) analysis was performed to assess clinical discriminative utility of 4D flow parameters, and area under the ROC curve (AUC) was used to characterize overall discriminative capability. Agreement between intra- and inter-observer measurements was assessed using intra-class correlation (ICC), paired t-tests and Bland–Altman plots. Statistical significance was set at *P* < 0.05.

## Results

### Participant characteristics and myocardial function

Demographic and clinical data are tabulated and compared in Table 2. The median [IQR] ages for healthy controls and rTOF subjects were 30 [22, 42] and 29 [21, 41] years, respectively. There were no significant differences in body surface area (BSA), heart rate, LV mass index, LVEDV index, LVESV index, LV stroke volume index, and LV ejection fraction (LVEF) between the two groups. All rTOF subjects were in sinus rhythm during both CMR and CPET. As expected, rTOF patients had significantly larger RV volumes and lower RVEF compared with healthy controls. Mean PRF among rTOF patients was 44%. Seven rTOF patients had moderate-to-severe TR. Forty-three (68%) rTOF patients had RVOT dyskinesia and RV restrictive physiology. rTOF patients had significant larger PVA diameter than healthy controls (2.7 ± 0.6 cm vs. 2.2 ± 0.3 cm, *P* < 0.001), and significantly higher PWV (3.3 ± 1.9 m/s vs. 1.9 ± 0.8 m/s, *P* < 0.01). rTOF patients had significantly higher inter-ventricular mechanical synchrony indexes than healthy controls with time to maximal displacement consistently longer in the RV free wall than LV lateral wall (Additional file 2: Figure S1(A)).

**Table 2:** Demographic comparison between healthy controls and repaired tetralogy of Fallot (rTOF)

	Healthy Control (n = 63)	rTOF (n = 63)	*P*
Demographics			
Age at CMR, yrs	30 [22, 42]	29 [21, 41]	0.695
Age at primary repair, yrs*	–	3 [1.25, 7.25]	–
Time after primary repair, yrs*	–	25 [20, 33]	–
Gender, M/F, n	29/34	29/34	1.000
Height, cm	161 ± 14	159 ± 13	0.551
Weight, kg	58 ± 18	57 ± 18	0.966
Body surface area, m^2^	1.59 ± 0.31	1.58 ± 0.30	0.741
Body mass index, kg/m^2^	21.8 ± 4.5	22.1 ± 5.5	0.729
Heart rate, bpm	75 ± 14	77 ± 12	0.483
Tricuspid regurgitation			
No	63 (100%)	28 (44%)	–
Mild	–	28 (44%)	–
Moderate to severe	–	7 (12%)	–
Numbers with re-intervention	–	17 (27%)	–
Pulmonary valve replacement	–	1 (2%)	–
Types of repair^†^			–
Transannular patch	–	45 (71%)	–
Valve-sparing	–	Unknown	–
Conduit	–	Unknown	–
Right ventricular outflow tract dyskinesia	–	43 (68%)	–
Restrictive physiology	–	43 (68%)	–
LV function			
LV mass index, g/m^2^	41 ± 8	41 ± 11	0.998
LV end-diastolic volume index, ml/m^2^	79 ± 13	78 ± 16	0.850
LV end-systolic volume index, ml/m^2^	33 ± 7	34 ± 11	0.496
LV stroke volume index, ml/m^2^	46 ± 8	44 ± 9	0.306
LV ejection fraction, %	58 ± 5	57 ± 7	0.242
RV function			
RV end-diastolic volume index, ml/m^2^	77 ± 14	133 ± 31	** < 0.001**
RV end-systolic volume index, ml/m^2^	36 ± 9	73 ± 23	** < 0.001**
RV stroke volume index, ml/m^2^	41 ± 7	60 ± 15	** < 0.001**
RV ejection fraction, %	54 ± 6	46 ± 8	** < 0.001**
RVEDV/LVEDV ratio	0.99 ± 0.12	1.75 ± 0.49	** < 0.001**
Pulmonary regurgitant volume, ml	–	44 ± 25	–
Pulmonary regurgitant fraction, %	–	43.5 ± 16.6	–
Pulmonary valve annulus diameter, cm	2.2 ± 0.3	2.7 ± 0.6	** < 0.001**
Pulse wave velocity, m/s	1.9 ± 0.8	3.3 ± 1.9	** < 0.01**

Data are presented as median [IQR], mean ± SD or n (%)

*CMR* cardiovascular magnetic resonance, *IQR* interquartile range, *LV* left ventricle, *LVEDV* left ventricular end-diastolic volume,  *RV* right ventricle, *RVEDV* right ventricular end-diastolic volume, *SD* standard deviation

*Age of primary repair data missing in 5 rTOF; ^†^Type of repair data missing in 18 rTOF patients

### Changes in flow components and kinetic energy profiles

**Fig. 2:**
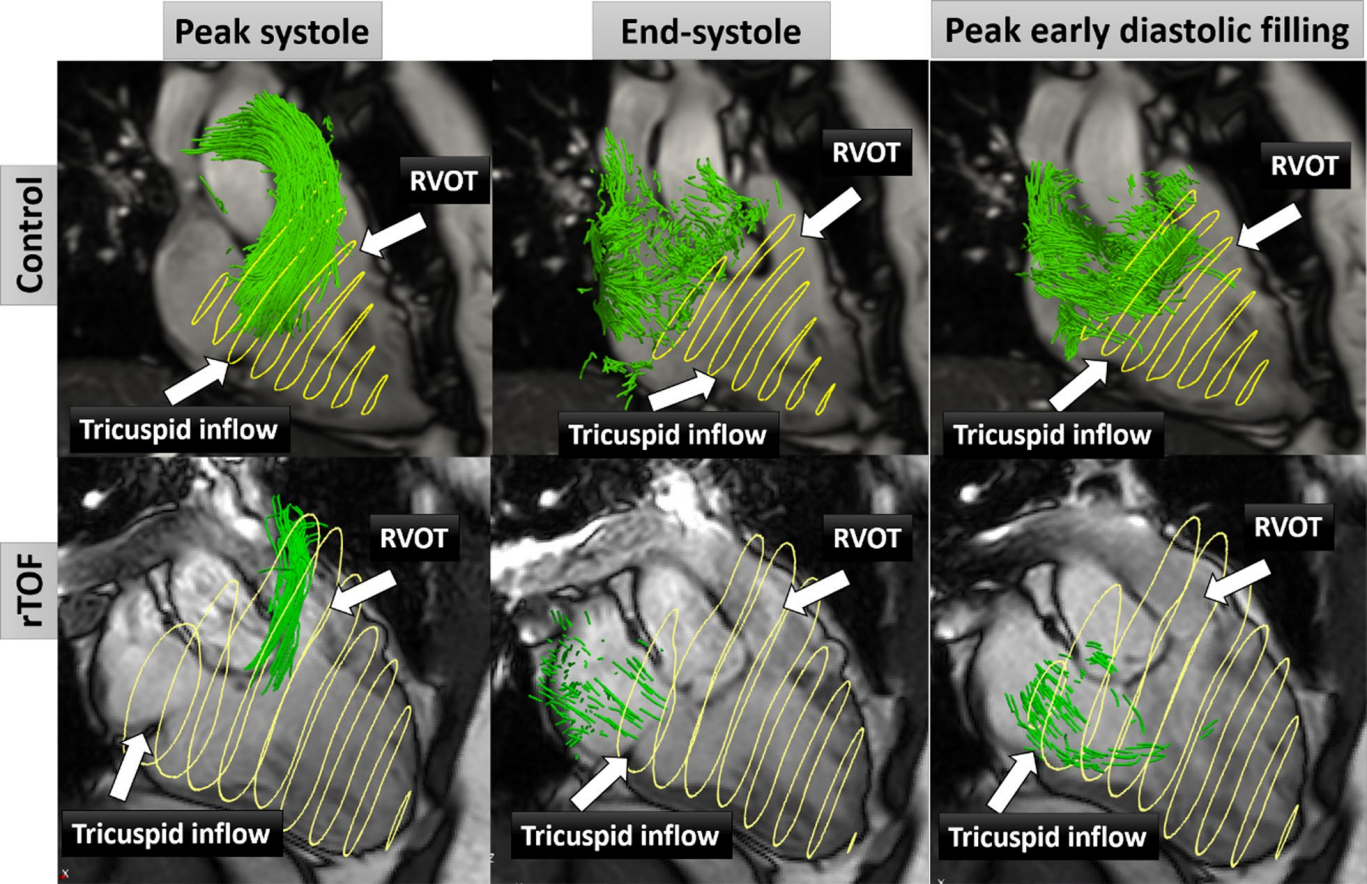
Right ventricle (RV) direct flow (green) using particle tracing at peak systole, end-systole and peak early diastolic filling phases in a 30-year-old healthy subject (first row) and a 28-year-old repaired tetralogy of Fallot (rTOF) patient (second row) with respective RV direct flow of 39% and 19%. Yellow circles denote the RV contours from stacks of short axis views. *RVOT* RV outflow tract

RV direct flow at peak systole, end-systole and peak early diastolic filling for one example each of rTOF patient and healthy subject are shown in Fig. 2. In the RV, rTOF patients had significantly reduced direct flow and increased delayed ejection flow, residual volume, RV peak systolic, average systolic, peak E-wave KEi_EDV_ and KE discordance compared with healthy controls (all *P* < 0.01, Table 3), whereas in the LV, rTOF patients had significantly higher retained inflow and peak E-wave KEi_EDV_ (both *P* < 0.01, Table 3). Moreover, rTOF patients had significantly higher inter-ventricular flow synchrony indexes (44 [31, 67] ms vs. 23 [0, 31] ms, *P* < 0.05) (Additional file 2: Figure S1(B)). There was no significant impact of severity of TR, presence of RVOT dyskinesia and inter-ventricular mechanical dyssynchrony on 4D flow CMR parameters except TR severity on RV peak systolic KEi_EDV_ (Additional file 3: Table S1). There was no significant impact of presence of RV restrictive physiology on 4D flow CMR parameters except RV direct flow, RV peak E-wave KEi_EDV_ and KE discordance (Additional file 3: Table S1). Only RV peak E-wave KEi_EDV_ positively correlated with PVA diameter (R = 0.358), PRF (R = 0.465) and PRV (R = 0.496), and KE discordance negatively correlated with PVA diameter (R = − 0.343) (Additional file 4: Table S2).

**Table 3:** Comparison of 4D flow and CPET parameters between healthy controls and repaired tetralogy of Fallot (rTOF)

	Healthy Control (n = 63)	rTOF (n = 63)	P*
Left ventricle (LV)			
Direct flow, %	33 (11)	32 (10)	0.304
Retained inflow, %	16 (6)	18 (7)	**0.005**
Delayed ejection flow, %	18 (5)	17 (7)	0.145
Residual volume, %	33 (8)	31 (10)	0.495
Peak systolic KEi_EDV_, µJ/ml	17.7 (5.9)	15.8 (8.5)	0.143
Average systolic KEi_EDV_, µJ/ml	10.0 (2.8)	8.7 (5.4)	0.233
Peak E-wave KEi_EDV_, µJ/ml	29.2 (11.1)	34.9 (21.9)	**0.006**
Right ventricle (RV)			
Direct flow, %	35 (8)	25 (10)	** < 0.001**
Retained inflow, %	16 (5)	17 (6)	0.526
Delayed ejection flow, %	17 (6)	21 (7)	** < 0.001**
Residual volume, %	31 (9)	39 (10)	** < 0.001**
Peak systolic KEi_EDV_, µJ/ml	23.3 (9.3)	29.9 (20.6)	**0.001**
Average systolic KEi_EDV_, µJ/ml	13.2 (4.6)	16.8 (8.7)	** < 0.001**
Peak E-wave KEi_EDV_, µJ/ml	14.8 (7.7)	29.8 (19.3)	** < 0.001**
KE discordance	1.27 (0.39)	1.79 (1.03)	** < 0.001**
Cardiopulmonary exercise testing^†^			
VE, l/min	42.5 (32.8)	32.8 (10.1)	**0.001**
Metabolic equivalents	6.8 (3.5)	5.1 (1.8)	** < 0.001**
Peak VO_2_, ml/kg/min	23.8 (12.4)	18.0 (7.3)	** < 0.001**
% predicted peak VO_2_, %	87 (43)	68 (23)	** < 0.001**
Anaerobic threshold, %	53 (16)	44 (15)	** < 0.001**
Heart rate reserve, %	13 (14)	26 (13)	** < 0.001**
VE/VCO_2_ slope	26 (4)	27 (5)	0.061

Data are presented as median (IQR), IQR = 75th percentile–25th percentile. *P value from Mann–Whitney U-Test

*CPET* cardiopulmonary exercise testing, *EDV* end-diastolic volume, *IQR* interquartile range, *KE* kinetic energy, *KE discordance* RV/LV systolic KEi_EDV_, *KEi*_*EDV*_ kinetic energy normalized to EDV, *LV* left ventricle, *RV* right ventricle, *VCO*_*2*_ carbon dioxide output, *VE* minute ventilation, *VO*_*2*_ oxygen uptake

^†^Only for 49 adult rTOF and 49 adult healthy controls

### Changes in cardiopulmonary exercise testing outcomes

Compared with healthy controls, rTOF patients had significantly lower VE, attained metabolic equivalents, peak VO_2_, % predicted peak VO_2_, anaerobic threshold, and higher heart rate reserve (all *P* < 0.001) (Table 3). No correlations were found between RVEDV index, RVESV index, RVEF and CPET outcomes. PRV was positively correlated with % predicted peak VO_2_ (R = 0.292, *P* = 0.042) and PRF was positively correlated with VE/VCO_2_ slope (R = 0.372, *P* = 0.008).

### Association of 4D flow parameters with RV remodelling index, function, and CPET outcomes

Correlation coefficients of 4D flow parameters and RV remodelling index (RVEDV/LVEDV ratio), RVEF and CPET parameters are shown in Table 4. RV direct flow correlated negatively with RVEDV/LVEDV ratio (*P* < 0.01) and positively with RVEF (*P* < 0.01), % predicted peak VO_2_ (*P* < 0.05) and anaerobic threshold (*P* < 0.05). RV residual volume correlated negatively with RVEF and positively with RVEDV/LVEDV ratio (both *P* < 0.01). For RV KE parameters, only RV peak E-wave KEi_EDV_ was positively correlated with RV remodelling, and negatively correlated with exercise capacity (peak VO_2_, metabolic equivalents, % predicted peak VO_2_ and anaerobic threshold), but the association persisted for RV remodelling but not for % predicted peak VO_2_ on multivariable analysis (Table 5).

**Table 4:** Pearson correlation coefficient R of 4D flow parameters and RV remodelling, RV function and CPET parameters in healthy controls and repaired tetralogy of Fallot (rTOF)

	RV direct flow, %	RV residual volume, %	LV direct flow, %	LV residual volume, %	RV peak systolic KEi_EDV_, µJ/ml	RV average systolic KEi_EDV_, µJ/ml	RV peak E-wave KEi_EDV_, µJ/ml	KE discordance
RVEDV/LVEDV ratio	− 0.582*	0.553*	− 0.207^†^	0.115	0.091	0.162	0.567*	0.159
RVEF, %	0.629*	− 0.594*	0.177^†^	− 0.252*	0.040	− 0.004	− 0.155	− 0.170
Peak VO_2_, ml/kg/min	0.118	− 0.069	0.132	0.075	− 0.029	− 0.034	− 0.242^†^	− 0.046
Metabolic equivalents	0.118	− 0.070	0.134	0.073	− 0.032	− 0.037	− 0.246^†^	− 0.049
% predicted peak VO_2_, %	0.214^†^	− 0.136	0.095	0.051	− 0.081	− 0.098	− 0.211^†^	− 0.094
Anaerobic threshold, %	0.208^†^	− 0.160	0.004	0.036	− 0.130	− 0.128	− 0.229^†^	− 0.123
VE/VCO_2_ slope	− 0.154	0.068	− 0.066	0.029	0.062	0.088	0.197	0.164

*CPET* cardiopulmonary exercise testing, *EDV* end-diastolic volume, *KE* kinetic energy, *KE discordance* RV/LV systolic KEi_EDV_, *KEi*_*EDV*_ kinetic energy normalized to EDV, *LV* left ventricular, *LVEDV* left ventricular end-diastolic volume, *RV* right ventricular, *RVEDV* right ventricular end-diastolic volume, *RVEF* right ventricular ejection fraction, *VCO*_*2*_ carbon dioxide output, *VE* minute ventilation, *VO*_*2*_ oxygen uptake

^*^Significance level < 0.01; ^†^Significance level < 0.05

**Table 5:** Univariate and multivariable linear regression analysis for determinants of RV dysfunction, RV dilation, and exercise capacity in healthy controls and repaired tetralogy of Fallot (rTOF)

	**Univariate analysis**		**Stepwise multivariable analysis**	
	**Coefficient (95% CI)**	***P* value**	**Coefficient (95% CI)**	***P* value**
Determinants of RVEF				
RV direct flow, %	0.574 (0.448, 0.700)	** < 0.001**	0.574 (0.448, 0.700)	** < 0.0001**
RV retained inflow, %	− 0.086 (− 0.476, 0.303)	0.661	–	
RV delayed ejection flow, %	− 0.077 (− 0.377, 0.222)	0.610	–	
RV residual volume, %	− 0.549 (− 0.681, − 0.416)	** < 0.001**	–	
RV peak systolic KEi_EDV_, µJ/ml	0.030 (− 0.102, 0.161)	0.655	–	
RV average systolic KEi_EDV_, µJ/ml	− 0.005 (− 0.229, 0.218)	0.963	–	
RV peak E-wave KEi_EDV_, µJ/ml	− 0.091 (− 0.194, 0.012)	0.083	–	
KE discordance	− 2.111 (− 4.285, 0.062)	0.057	–	
R-squared, multivariable				0.396
Determinants of RV remodelling index (RVEDV/LVEDV ratio)				
RVEF, %	− 0.025 (− 0.035, − 0.016)	** < 0.001**	–	
RV direct flow, %	− 0.033 (− 0.041, − 0.025)	** < 0.0001**	− 0.025 (− 0.032, − 0.017)	** < 0.001**
RV retained inflow, %	− 0.005 (− 0.029, 0.019)	0.664	–	
RV delayed ejection flow, %	0.010 (− 0.009, 0.028)	0.304	–	
RV residual volume, %	0.031 (0.023, 0.040)	** < 0.001**	–	
RV peak systolic KEi_EDV_, µJ/ml	0.004 (− 0.004, 0.012)	0.310	–	
RV average systolic KEi_EDV_, µJ/ml	0.013 (− 0.001, 0.026)	0.071	–	
RV peak E-wave KEi_EDV_, µJ/ml	0.021 (0.015, 0.026)	** < 0.001**	0.015 (0.010, 0.020)	** < 0.001**
KE discordance	0.122 (− 0.012, 0.256)	0.075	–	
R-squared, multivariable				0.492
Determinants of exercise capacity (% predicted peak VO_2_)*				
RVEF, %	0.386 (− 0.341, 1.113)	0.295	–	
RV direct flow, %	0.626 (0.048, 1.203)	**0.034**	0.626 (0.048, 1.203)	**0.034**
RV retained inflow, %	− 0.919 (− 2.321, 0.484)	0.197	–	
RV delayed ejection flow, %	− 0.361 (− 1.448, 0.726)	0.512	–	
RV residual volume, %	− 0.431 (− 1.608, 0.207)	0.183	–	
RV peak systolic KEi_EDV_, µJ/ml	− 0.185 (− 0.651, 0.280)	0.431	–	
RV average systolic KEi_EDV_, µJ/ml	− 0.398 (− 1.219, 0.423)	0.338	–	
RV peak E-wave KEi_EDV_, µJ/ml	− 0.362 (− 0.702, − 0.022)	**0.037**	–	
KE discordance	− 4.138 (− 13.049, 4.773)	0.359	–	
LV direct flow, %	0.331 (− 0.375, 0.036)	0.354	–	
LV retained inflow, %	− 0.631 (− 1.626, 0.363)	0.210	–	
LV delayed ejection flow, %	− 0.399 (− 1.463, 0.666)	0.459	–	
LV residual volume, %	0.184 (− 0.541, 0.908)	0.616	–	
R-squared, multivariable				0.046

*CI* confidence interval, *EDV* end-diastolic volume, *KE* kinetic energy, *KE discordance* RV/LV systolic KEi_EDV_, *KEi*_*EDV*_ kinetic energy normalized to EDV, *LV* left ventricle, *LVEDV* left ventricular end-diastolic volume, *RV* right ventricle, *RVEDV* right ventricular end-diastolic volume, *RVEF* right ventricular ejection fraction, *VO*_*2*_ oxygen uptake

^*^Only for 49 adult rTOF and 49 adult healthy controls

**Fig. 3:**
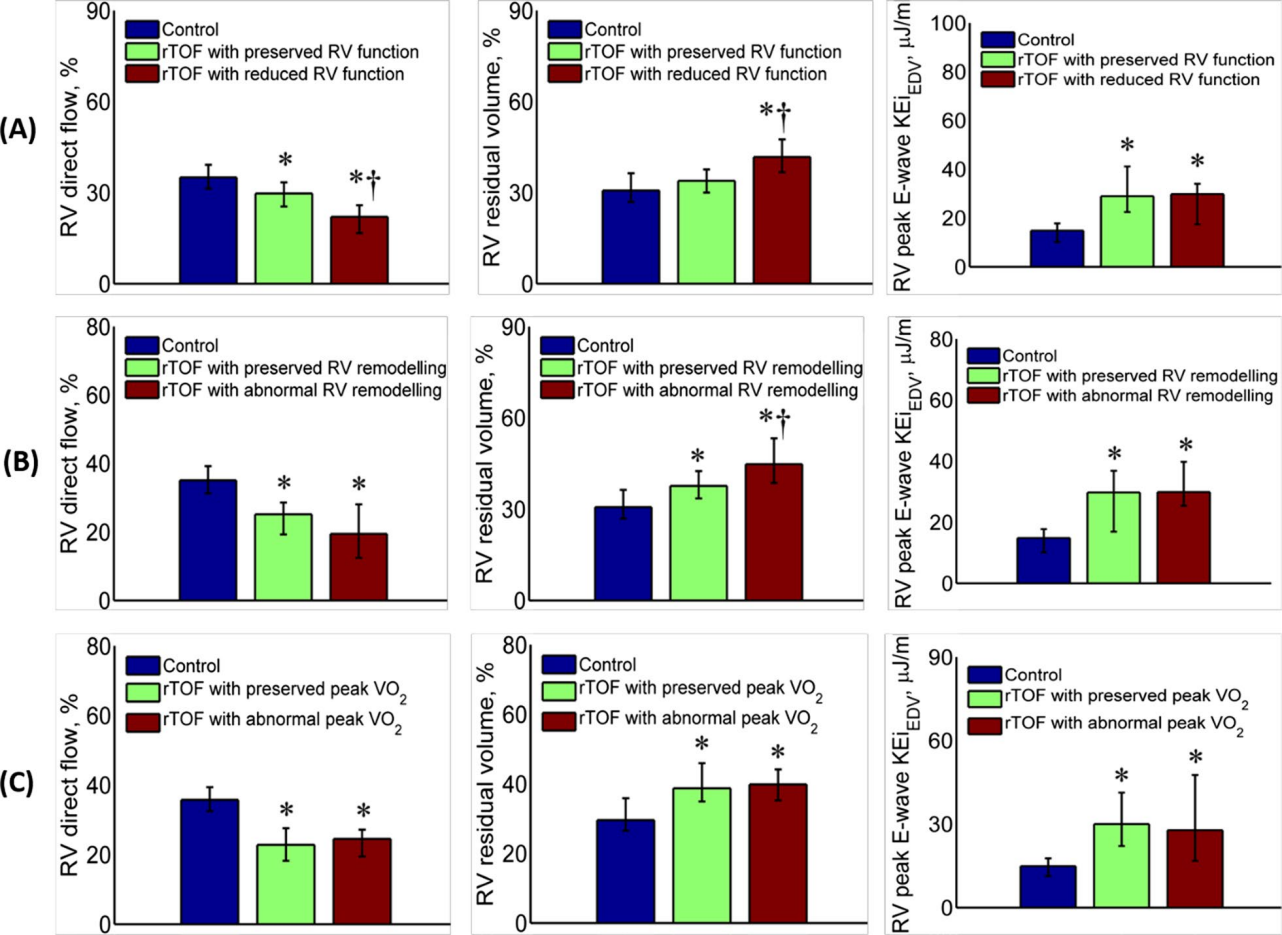
Differences in 4D flow right ventricular (RV) parameters according to RV function; RV remodelling; and peak oxygen uptake (VO_2_). RV direct flow (left), RV residual volume (middle) and peak E-wave KEi_EDV_ (right) are presented (**A**) among healthy controls, rTOF with preserved RV function, and rTOF with reduced RV function; (**B**) among healthy controls, rTOF with preserved RV remodelling and rTOF with abnormal RV remodelling; (**C**) among healthy controls, rTOF with preserved peak VO_2_ and rTOF with abnormal peak VO_2_. *P < 0.05 compared with healthy controls; ^†^*P* < 0.05 compared with rTOF with preserved RV function, and rTOF with preserved RV remodelling, respectively. Error bars denote median—25th percentile (lower) and 75th percentile—median (upper). *KEi*_*EDV*_ kinetic energy normalized to end-diastolic volume (EDV), *rTOF* repaired tetralogy of Fallot

A progressive decrease in RV direct flow was observed with decreasing RVEF, and rTOF patients with reduced RV function had significantly reduced RV direct flow and increased RV residual volume compared with healthy controls and rTOF with preserved RV function (Fig. 3A). Compared with healthy controls, RV direct flow, residual volume, and peak E-wave KEi_EDV_ differed significantly between rTOF patient with preserved RV remodelling (RVEDV/LVEDV ratio > 2) vs. abnormal RV remodelling (RVEDV/LVEDV ratio ≤ 2) (Fig. 3B) and with preserved peak VO_2_ (> 15 ml/kg/min) vs. abnormal peak VO_2_ (≤ 15 ml/kg/min) (Fig. 3C).

On multivariate analysis, RV direct flow was independently associated with RV dysfunction ($\widehat{\beta }$=0.574, *P* < 0.001) and was the only sensitive marker for predicting CPET outcome in adult rTOF and adult healthy controls ($\widehat{\beta }$=0.626, *P* = 0.034). RV direct flow ($\widehat{\beta }$=− 0.025, *P* < 0.001) and RV peak E-wave KEi_EDV_ ($\widehat{\beta }$=0.015, *P* < 0.001) were independently associated with RV remodelling (Table 5).

**Fig. 4:**
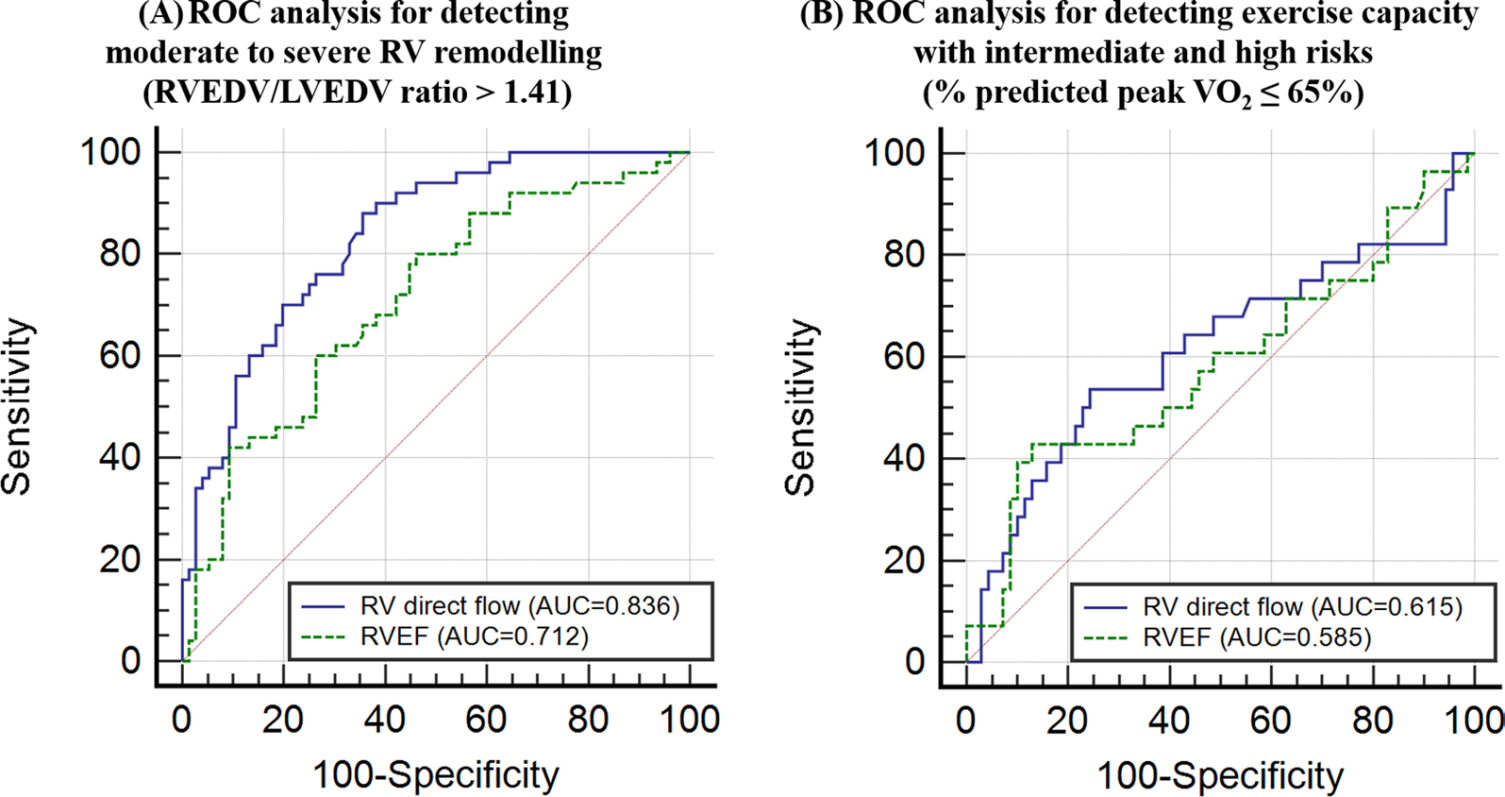
Utility of right ventricular (RV) direct flow and RV ejection fraction (RVEF) to detect (**A**) moderate to severe RV remodelling (RVEDV/LVEDV ratio > 1.41) (**B**) exercise capacity with intermediate and high risks (% predicted peak VO_2_ < 65%). *AUC* area under ROC curve, *EDV* end-diastolic volume, *ROC* receiver operating characteristic, *LV* left ventricle, *LVEDV* left ventricular end-diastolic volume, *RVEDV* right ventricular end-diastolic volume, *VO*_*2*_ oxygen uptake

ROC analysis demonstrated that RV direct flow (AUC = 0.836, sensitivity = 88%, specificity = 65%, cut-off = 31%) had better discrimination for moderate-to-severe RV remodelling defined as RVEDV/LVEDV ratio > 1.41 than RVEF (AUC = 0.712) (Fig. 4A). In addition, RV direct flow (AUC = 0.615, sensitivity = 54%, specificity = 76%, cut-off = 26%) had better discrimination than RVEF (AUC = 0.585) for intermediate and high risks based on exercise capacity (% predicted peak VO_2_ < 65%) (Fig. 4B).

### Reproducibility

**Fig. 5:**
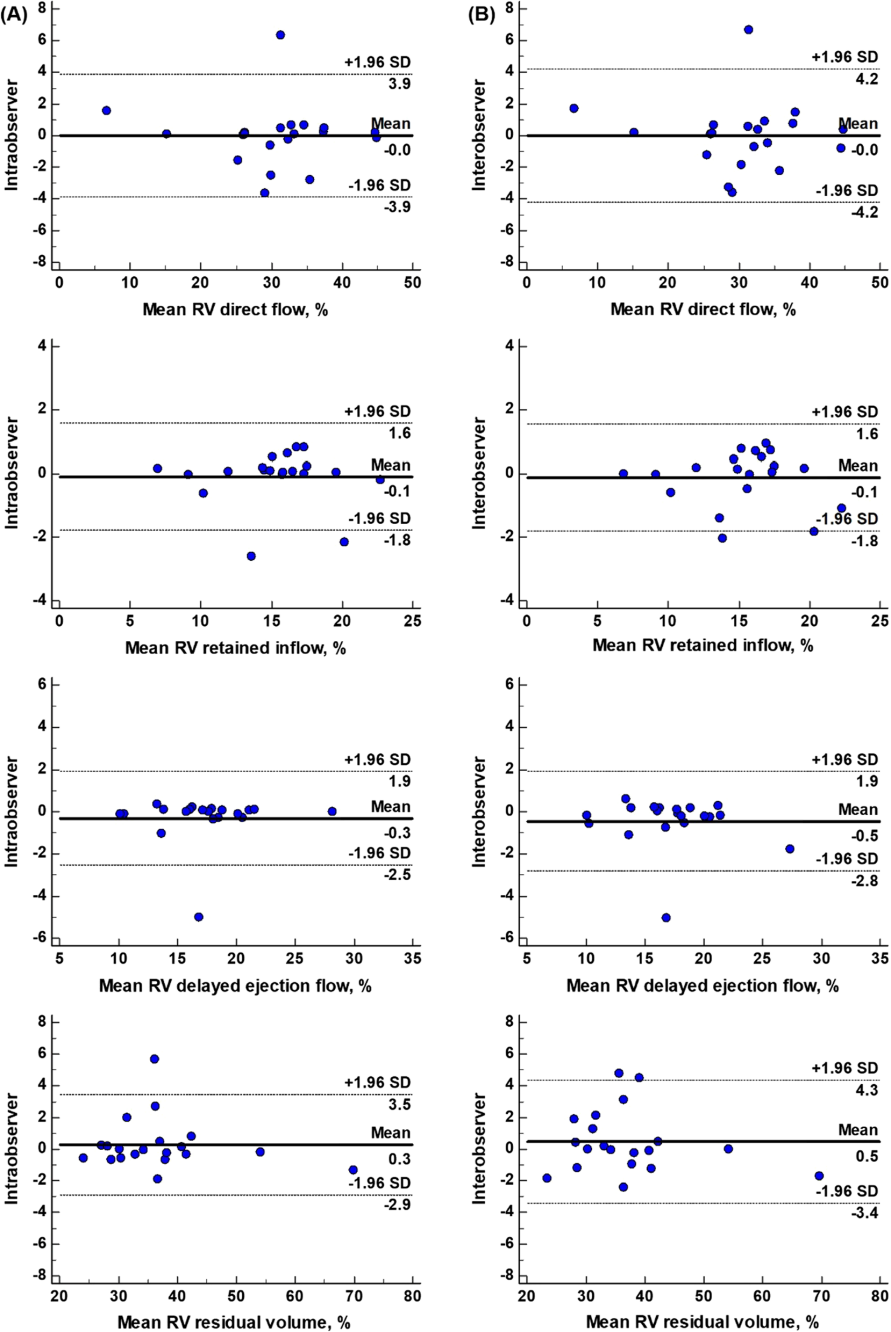
Reproducibility of right ventricular (RV) 4D flow components. (**A**) Bland–Altman analysis of intra-observer repeated measurements of RV direct flow (first row), RV retained inflow (second row), RV delayed ejection flow (third row) and RV residual volume (last row); (**B**) Bland–Altman analysis of inter-observer repeated measurements of RV direct flow (first row), RV retained inflow (second row), RV delayed ejection flow (third row) and RV residual volume (last row)

The reproducibility results of RV 4D flow components for 10 healthy controls and 10 rTOF are tabulated in Table 6. Both intra- and interobserver had excellent intraclass correlation coefficients (all > 0.97, *P* < 0.001). Mean intra- and interobserver differences measurements were small with good limits of agreement (Table 6). Bland–Altman plots of intra- and inter-observer measurements were given in Fig. 5. Coefficients of variation for inter-observer reproducibility were 4.9%, 3.9%, 5.2%, 3.8% for direct flow, retained inflow, delayed ejection flow and residual volume, respectively; and for intra-observer reproducibility 4.5%, 3.9%, 4.7% and 3.1%, respectively.

**Table 6:** Intra- and interobserver agreement

	Mean ± difference	*P*	ICC (95% CI)	*P*	CV
Intraobserver					
RV direct flow, %	− 0.013 ± 1.985	0.977	0.987 (0.967, 0.995)	< 0.001	4.5
RV retained inflow, %	− 0.088 ± 0.855	0.653	0.987 (0.967, 0.995)	< 0.001	3.9
RV delayed ejection flow, %	− 0.304 ± 1.139	0.248	0.981 (0.951, 0992)	< 0.001	4.7
RV residual volume, %	0.275 ± 1.624	0.458	0.994 (0.984, 0.998)	< 0.001	3.1
Interobserver					
RV direct flow, %	− 0.011 ± 2.145	0.983	0.985 (0.962, 0.994)	< 0.001	4.9
RV retained inflow, %	− 0.123 ± 0.860	0.525	0.987 (0.967, 0.995)	< 0.001	3.9
RV delayed ejection flow, %	− 0.453 ± 1.206	0.110	0.977 (0.943, 0991)	< 0.001	5.2
RV residual volume, %	0.456 ± 1.980	0.316	0.991 (0.976, 0.996)	< 0.001	3.8

Mean ± difference between repeated measures and significance were tested with a paired Student t-test and agreement using intra-class correlation coefficient (ICC). *CI* confidence interval, *CV* coefficients of variation, *RV* right ventricle

## Discussion

This large-scale multicenter study aimed to investigate the potential role of 4D flow CMR in a specific pathophysiologic state: PR-induced RV volume overload in rTOF. Compared with healthy controls, we observed lower RV direct flow in rTOF patients. Both RV direct and residual flows exhibited significant correlations with RV remodelling index, RV functional parameters and CPET outcomes. Finally, we demonstrated RV direct flow to be the most discriminative marker for predicting adverse RV remodelling, RV dysfunction and impaired exercise capacity compared with standard RV volume and RVEF measurements.

### Relationship between RV direct flow and kinetic energy and differences

Flow component analysis sheds light on the efficiency of blood flow transit [39] and is especially illuminating in our rTOF study population with significant pulmonary regurgitation (mean PRF 44%), which both induces and co-exists with RV modelling. Comparing rTOF subjects with healthy controls, blood flow transit is inefficient despite higher stroke volume as a smaller proportion of flow transits the RV per cardiac cycle (direct flow 25% vs 35%, *P* < 0.001) and a higher proportion is being recirculated within the chamber (residual volume 39% vs 31%, *P* < 0.001). KE represents work performed by the RV and is expressed as the product of blood volume and the square of velocity. KE can be segmented temporally into different phases of the cardiac cycle as in the current study and others [40], or more granularly into the different flow components at different times, e.g., LV end-diastolic direct flow KE [33]. In our study, segmenting of KE of all voxels into time-resolved parcels allows dissection into the work done by the RV at various phases of the cardiac cycle as KE of the inflowing blood is transferred into motion of the blood already residing in the RV, converted into potential energy stored in the myocardium, or dissipated as heat. We observed higher KE in rTOF compared with healthy controls, which can probably be explained in part as compensation for PR. Our results corroborate the findings of Robinson et al., who reported in paediatric rTOF patients a non-linear correlation between RV diastolic KE and RVEDV index, a traditional metric of disease progression [40].

RV direct flow, the effective propagation of the tricuspid inflow diastolic vortex into the RVOT, which can then be ejected in systole. This flow property has been studied in prior studies, both in-vivo and in-vitro (by computational fluid dynamics and particle velocimetry), in both normal healthy controls and rTOF patients [41–45]. Moreover, Michail and his colleague [45] showed that PR significantly altered the tricuspid inflow during diastolic phase with increasing viscous energy dissipation in the RV in an in-vitro study. These may explain why RV direct flow significantly reduced in rTOF patients, as demonstrated in our study.

In our study, RV direct flow discriminates for RVEF and RV remodelling index in a multiple linear regression model, which supports its utility as a marker of disease progression and potential of response to recommended or experimental therapies. The association of RV direct flow with exercise capacity is more modest but bears closer examination as there has been a dearth of longitudinal studies on exercise capacity in rTOF, which belies its influence on survival in related conditions like left heart failure [46]. In the largest cross-sectional survey of exercise capacity in rTOF involving 586 patients aged 6 to 63 years, there was a worrisome accelerated decline with age compared to controls [47], which underscores the case for vigilant surveillance. Persistence of the association between RV direct flow and exercise capacity even after accounting for RVEF warrants further longitudinal study investigating its prognostic utility.

### Relation to earlier studies

Our findings support the utility of 4D flow CMR-assessed RV direct flow component as a sensitive imaging parameter in the surveillance of paediatric and adult rTOF patients. In rTOF, RV dilation demonstrated little correlation whereas RV direct flow component was related to exercise capacity, which is a reflection of functional health status. Traditional RV function parameters such as RVEF are less sensitive and can remain preserved in both paediatric and adult rTOF patients even in the presence of RV myocardial deformation impairment [48, 49]. CPET provides valuable comprehensive information on peak oxygen uptake and minute ventilation/carbon dioxide production, and is considered a surrogate clinical endpoint. Our study demonstrated that RV direct flow is a more accurate predictor of peak VO_2_ than the conventional RV volume and function measurements. Future incorporation of these 4D flow CMR imaging parameters may redefine risk prognostication for rTOF patients and potentially be used to guide the need for and timing of intervention (i.e., pulmonary valve replacement).

Ventricular KE measurement is a novel imaging parameter in patients with rTOF. Geiger and Francois previously studied flow patterns and vorticity in rTOF [13, 50] and found altered RV vortex structure. Recently, Jeong et al. provided quantitative intra-cardiac flow KE measurements and showed that rTOF exhibited numerically higher peak systolic KE in both RV and LV than in healthy subjects but did not achieve statistical significance, likely due to the small study sample size (10 rTOF and 9 healthy subjects) [14]. Similarly, Sjoberg et al. found higher RV KE in rTOF than in healthy subjects [15]. Robinson et al. also observed higher diastolic RV KE indexed to segmentation volume in children with rTOF (n = 21) than healthy controls (n = 24) [40], and a non-linear relationship between diastolic RV KE indexed to BSA with RVEDV indexed to BSA. We were able to demonstrate statistically significantly higher KE measurements in rTOF vs. healthy controls in the largest 4D flow CMR rTOF cohort to date. Collectively, these findings suggest that greater ventricular KE is necessary to generate flow in the pulmonary and aortic circulation in rTOF.

### Impact of loading conditions on RV flow component and kinetic energy parameters

Within any group of rTOF patients, there is likely to be a wide range of loading conditions, which comprise a mix of preload (from PR and/or TR) and afterload (from valvar and/or stenosis/hypoplasia of main or pulmonary arteries). There is significant impact of PRF and PRV on RV diastolic peak E-wave KEi_EDV_ (R  =  0.465, *P* <  0.001; and R = 0.496, *P* < 0.001, respectively) but not the relative RV flow components (Additional file 4: Table S2). The former observation is consistent with the literature. Fredriksson et al. [51] stratified 27 rTOF patients into high (>11%) and low (≤ 11%) PRF groups, and observed turbulent KE (associated with diastolic PR flow) to be significantly higher in the former. The peak total RV turbulent KE (in the diastolic phase with the highest turbulent KE) was the strongest predictor of indexed RVEDV (R^2^ =  0.47, *P* = 0.002) in multivariable regression analyses. In Robinson et al. [40], RV diastolic KE was positively linearly correlated to PRF (R^2^  = 0.54, *P* < 0.01) among 21 rTOF patients. While there was also fair correlation between diastolic and systolic RV KE suggesting the increased RV systolic energy expenditure may be compensating for the PR—the correlation between RV systolic KE and PRF in rTOF was not reported. There is sparse literature on flow component analysis per se in rTOF. A recent systematic review of 4D flow CMR in rTOF [52] identified 26 studies, but none on 4D flow components. Hence, our findings of the lack of impact of PR on flow components are original. Of note, the significant associations between RV direct flow and RV remodelling index as well as RVEF (Tables 4 and 5) suggest that the morphological and functional consequences of PR rather than its severity at a single point in time dominate RV direct flow in rTOF, which is an interesting observation that is best confirmed with longitudinal assessment.

In this cohort, TR severity, pulmonary valve annulus dimension, main pulmonary artery stiffness (as assessed by PWV) had no significant effects on 4D flow components in rTOF. Second, the degree of dyskinesia of the resected/scarred or patched RVOT anterior wall did not significantly affect RV KE and the direct/retained/delayed flow parameters. Third, 43/63 rTOF patients (68%) had restrictive RV physiology. This relatively high proportion may be attributable to the preponderance of significant PR among our study patients (mean PRF 44%, 78% with PRF > 30%) and the long interval between primary repair and study assessment (median 25 years). Often, in the situation of high RV afterload with normal LV afterload, inter-ventricular dyssynchrony occurs since the end-systole timing of RV is delayed because the RV systolic phase is prolonged—sometimes 80–100 ms after LV end-systole. However, inter-ventricular dyssynchrony had no significant impact on flow components and KE parameters in rTOF patients.

### Study implication

The result findings are akin the 4D flow studies of LV heart disease. Among 10 dilated cardiomyopathy patients with well-compensated mild heart failure, 4D flow CMR revealed impaired preservation of LV end-diastolic direct flow KE despite similar LV stroke volumes as controls [33]. In another study involving 22 subjects with primary LV disease, RV dysfunction was demonstrated by RV impaired direct flow and end-diastolic KE but not standard CMR measures [53]. In the current study of 63 rTOF subjects (mean PRF 44%), RV direct flow proportion was significantly reduced compared with healthy controls (25% vs. 35%, *P* < 0.001) and was independently associated with RV remodelling and, to a lesser extent, exercise capacity. 4D flow CMR provides unique insights into chamber flow dynamics and work in the RV volume overload state, and our findings point to RV direct flow as an indicator of RV maladaptation. Its modest but significant correlation with exercise capacity suggests potential prognostic utility that warrants further longitudinal investigation. Pulmonary valve replacement (PVR) reduces RV size and reserves remodelling and could potentially restore the natural flow dynamic and increase direct flow.

## Limitations

4D RV flow components were correlated with a subject’s functional capacity. These relationships of blood flow component alterations with RV remodelling may elucidate additional mechanisms. However, this study is limited by its design as a cross-sectional observational study, which precludes the inference of causality in relationships. Secondly, the study dataset of the present study was acquired on using multi-vendor scanners (Philips, Siemens and GE) at different field strengths (3.0 T and 1.5 T) and using under different 4D flow CMR protocols. We standardized the CMR image acquisition procedures as much as possible to mitigate technical differences, and all were consistent with current recent consensus recommendations [19]. Each center acquired the data with the accepted spatial resolution and also the temporal resolution was not much different. Both spatial and temporal resolution are in line with the published recommendations for cardiac 4D flow [20]. Improvement in spatial and temporal resolution could result in more accurate particle tracing results, provided that the noise level of the data remains the same. Similarly, the post-processing steps of LV and RV segmentation, volumetric quantification, flow component and kinetic energy analyses were protocolized according to the standard recommendation. Thirdly, this study has a relatively modest sample size, which limits its statistical power. Nevertheless, the study was able to clearly demonstrate RV direct flow component as a superior predictor of adverse RV remodelling and impaired exercise capacity, a surrogate of clinical prognosis, compared with conventional volume and function parameters. None of our paediatric rTOF patients underwent CPET due to logistical reasons. PVR reduces RV size and reserves remodelling and could potentially restore the natural flow dynamic and increase direct flow. To what extent PVR impacts 4D flow parameters would be interested to be studied in the near future. Lastly, future studies with longitudinal follow-up data are needed to investigate the clinical prognostic measurements of 4D flow parameters, although clinical outcomes from CPET have been examined.

## Conclusions

Our data demonstrate that the RV direct flow component is sensitive in predicting RV remodelling, dysfunction and, to a lesser extent, exercise intolerance in subjects with RV volume loading in the context of rTOF. Hence, 4D flow CMR parameters are proposed as novel imaging markers that provide additional discriminatory information that may serve as useful surrogate endpoints in clinical follow-up as well as in interventional studies of rTOF. The results of this study suggest that 4D flow CMR parameters may play a valuable predictive role in studies of other physiologic types of right heart failure.

## Supplementary Information

**Additional file 1: **Movies showing four-chamber views with right ventricle (RV) four flow components using particle tracing in a 30-year-old normal subject and a 28-year-old repaired tetralogy of Fallot (rTOF) patient. Yellow circles denote the RV contours from stacks of short axis views. Color legend: green (RV direct flow), yellow (RV retained inflow), blue (RV delayed ejection flow), red (RV residual volume).

**Additional file 2: Figure S1. (A)** Difference in time to maximal displacement between right ventricle (RV) free wall and left ventricle (LV) lateral wall (Time difference = RV $-$ LV); (B) Difference in time to minimal kinetic energy normalized to end-diastolic volume during systole between right ventricle (RV) and left ventricle (LV) (Time difference = RV $-$ LV) for healthy control (left) and rTOF (right).

**Additional file 3: Table S1.** 4D flow parameters in repaired tetralogy of Fallot (rTOF) with no or mild tricuspid regurgitation (TR) vs. with moderate to severe TR, without RVOT dyskinesia versus with RVOT dyskinesia, without inter-ventricular mechanical dyssynchrony versus with inter-ventricular dyssynchrony, without restrictive physiology versus with restrictive physiology.

**Additional file 4: Table S2.** Correlation between 4D flow parameters and PV annulus diameter, MPA stiffness based on PWV index and pulmonary regurgitation parameters in repaired tetralogy of Fallot (rTOF).

## Data Availability

The datasets used and/or analysed during the current study are available from the corresponding author on reasonable request.

## Abbreviations

2D: Two-dimensional
4D: Four-dimensional
AUC: Area under ROC curves
BSA: Body surface area
CHD: Congenital heart disease
CI: Confidence interval
CMR: Cardiovascular magnetic resonance
CPET: Cardiopulmonary exercise testing
CV: Coefficients of variation
ECG: Electrocardiogram
EDV: End-diastolic volume
EF: Ejection fraction
EPI: Echo-planar imaging
ESV: End-systolic volume
GRAPPA: Generalized autocalibrating partially parallel acquisition
ICC: Intra-class correlation coefficient
IQR: Interquartile range
kat-ARC: K-adaptive-t autocalibrating reconstruction for Cartesian sampling
KE: Kinetic energy
KEi_EDV_: Kinetic energy normalized to end-diastolic volume
LV: Left ventricle/left ventricular
LVEDV: Left ventricular end-diastolic volume
LVEF: Left ventricular ejection fraction
LVESV: Left ventricular end-systolic volume
METS: Metabolic equivalents
MPA: Main pulmonary artery
PC: Phase-contrast
PR: Pulmonary regurgitation
PRF: Pulmonary regurgitant fraction
PRV: Pulmonary regurgitant volume
PVA: Pulmonary valve annulus
PVR: Pulmonary valve replacement
PV: Pulmonary valve
PWV: Pulse wave velocity
ROC: Receiver operating characteristic
rTOF: Repaired tetralogy of Fallot
RV: Right ventricle/right ventricular
RVEDV: Right ventricular end-diastolic volume
RVEF: Right ventricular ejection fraction
RVESV: Right ventricular end-systolic volume
RVOT: Right ventricular outflow tract
SD: Standard deviation
TE: Echo time
TOF: Tetralogy of Fallot
TR: Tricuspid regurgitation
VCO_2_: Carbon dioxide output
VE: Minute ventilation
VENC: Velocity encoding
VO_2_: Oxygen uptake
V_voxel_: Voxel volume
${v}_{\mathrm{voxel}}$: Velocity of the corresponding voxel
ρ_blood_: Blood density
