# Supplementary material for: Ventricular flow analysis and its association with exertional capacity in repaired tetralogy of Fallot: 4D flow cardiovascular magnetic resonance study
Source: J Cardiovasc Magn Reson. 2022 Jan 3;24:4. doi: 10.1186/s12968-021-00832-2 (PMC8722058; doi:10.1186/s12968-021-00832-2)
Supplement: Supplementary file 2 — Additional file 2: Figure S1. (A) Difference in time to maximal displacement between right ventricle (RV) free wall and left ventricle (LV) lateral wall (Time difference = RV $-$- LV); (B) Difference in time to minimal kinetic energy normalized to end-diastolic volume during systole between right ventricle (RV) and left ventricle (LV) (Time difference = RV $-$- LV) for healthy control (left) and rTOF (right). [file 12968_2021_832_MOESM2_ESM.docx]

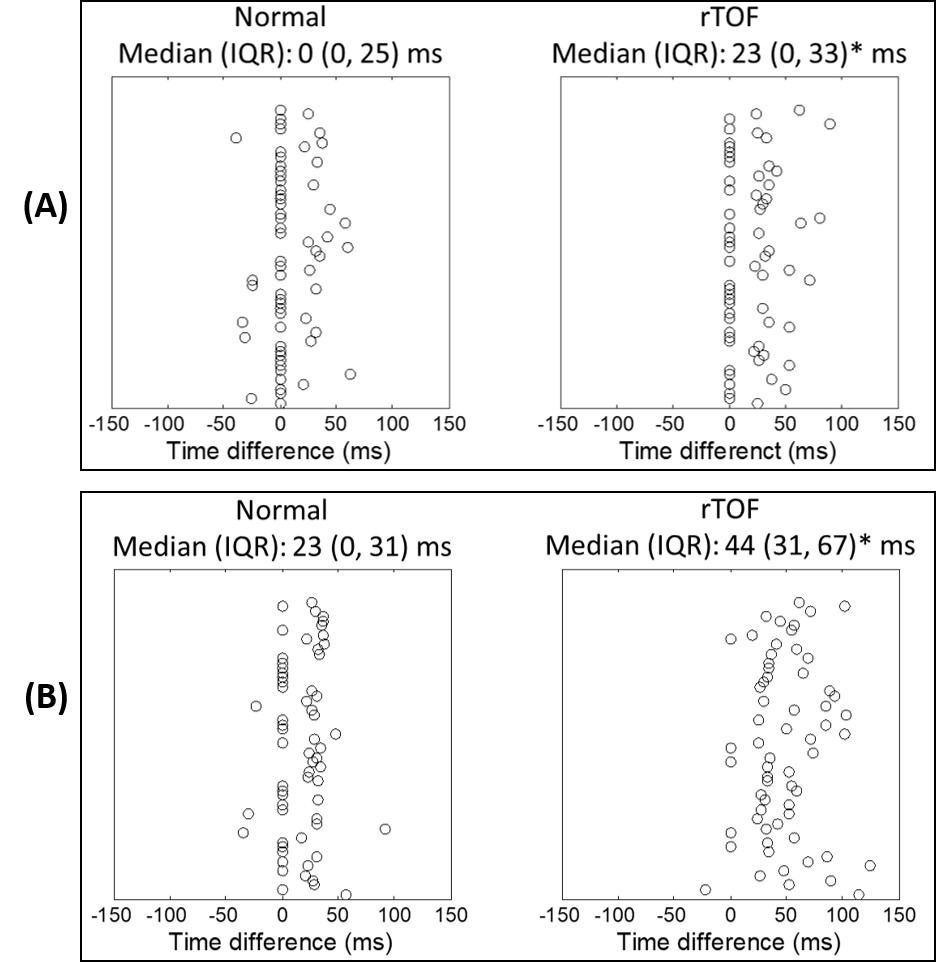


**Supplementary Figure 1. (A)** Difference in time to maximal displacement between right ventricle (RV) free wall and left ventricle (LV) lateral wall (Time difference = RV$-$LV); **(B)** Difference in time to minimal kinetic energy normalized to end-diastolic volume during systole between right ventricle (RV) and left ventricle (LV) (Time difference = RV$-$LV) for control (left) and rTOF (right). *IQR* interquartile range, *rTOF* repaired tetralogy of Fallot. *Significantly greater than controls.
