# Supplementary material for: Ventricular flow analysis and its association with exertional capacity in repaired tetralogy of Fallot: 4D flow cardiovascular magnetic resonance study
Source: J Cardiovasc Magn Reson. 2022 Jan 3;24:4. doi: 10.1186/s12968-021-00832-2 (PMC8722058; doi:10.1186/s12968-021-00832-2)
Supplement: Supplementary file 3 — Additional file 3: Table S1. 4D flow parameters in repaired tetralogy of Fallot (rTOF) with no or mild tricuspid regurgitation (TR) vs. with moderate to severe TR, without RVOT dyskinesia versus with RVOT dyskinesia, without inter-ventricular mechanical dyssynchrony versus with inter-ventricular dyssynchrony, without restrictive physiology versus with restrictive physiology. [file 12968_2021_832_MOESM3_ESM.docx]

**Table S1.** 4D flow parameters in repaired tetralogy of Fallot (rTOF) with no or mild tricuspid regurgitation (TR) vs. with moderate to severe TR, no RVOT dyskinesia versus with RVOT dyskinesia, no inter-ventricular mechanical dyssynchrony versus with inter-ventricular dyssynchrony, no restrictive physiology versus with restrictive physiology.

| **4D flow parameters** | **No or mild TR**  **(n=56)** | **Moderate to severe TR (n=7)** | **No RVOT dyskinesia (n=20)** | **With RVOT dyskinesia**  **(n=43)** | **No inter-ventricular mechanical dyssynchrony (n=54)**† | **With inter-ventricular mechanical dyssynchrony (n=9)**† | **No restrictive physiology**  **(n=20)** | **With restrictive physiology**  **(n=43)** |
| --- | --- | --- | --- | --- | --- | --- | --- | --- |
| RV direct flow, % | 25 (10) | 18 (14) | 25 (6) | 24 (13) | 25 (10) | 27 (12) | 21 (11) | 25 (11)* |
| RV retained inflow, % | 16 (5) | 18 (6) | 17 (7) | 16 (5) | 17 (6) | 17 (4) | 16 (4) | 17 (6) |
| RV delayed ejection flow, % | 21 (7) | 17 (9) | 20 (8) | 21 (7) | 21 (7) | 21 (6) | 20 (9) | 21 (6) |
| RV residual volume, % | 38 (10) | 39 (17) | 38 (17) | 39 (12) | 39 (10) | 39 (14) | 42 (18) | 38 (8) |
| RV peak systolic KEi_EDV_, µJ/ml | 30.3 (20.4) | 17.5 (15.8)* | 29.7 (17.2) | 30.0 (21.0) | 29.7 (20.8) | 30.3 (21.7) | 24.6 (23.8) | 30.8 (18.2) |
| RV average systolic KEi_EDV_, µJ/ml | 17.5 (9.0) | 14.4 (6.6) | 15.6 (7.0) | 17.6 (10.9) | 16.7 (8.3) | 17.6 (12.9) | 17.0 (9.4) | 16.8 (8.9) |
| RV peak E-wave KEi_EDV_, µJ/ml | 28.6 (19.6) | 32.4 (16.5) | 26.8 (18.3) | 30.0 (20.5) | 29.9 (18.0) | 29.8 (19.6) | 23.1 (15.1) | 30.5 (15.3)* |
| KE discordance | 1.80 (1.00) | 1.09 (0.84) | 1.79 (0.91) | 1.79 (1.05) | 1.79 (1.04) | 1.83 (0.95) | 1.18 (1.15) | 1.86 (0.89)* |

Data are presented as median (IQR), IQR = 75^th^ percentile – 25^th^ percentile. **P* value from Mann-Whitney U-Test.

*IQR* interquartile range, *KE* kinetic energy, *KE discordance* RV/LV systolic KEi_EDV_, *KEi_EDV_* kinetic energy normalized to end-diastolic volume (EDV), *LV* left ventricle, *PVR* pulmonary valve replacement, *RV* right ventricular, *RVOT* right ventricular outflow tract.

†Inter-ventricular dyssynchrony assessed by difference in time to maximal displacement between RV free wall and LV lateral wall (i.e. ≥44 ms by 95^th^ percentile from controls).
