# Supplementary material for: Ventricular flow analysis and its association with exertional capacity in repaired tetralogy of Fallot: 4D flow cardiovascular magnetic resonance study
Source: J Cardiovasc Magn Reson. 2022 Jan 3;24:4. doi: 10.1186/s12968-021-00832-2 (PMC8722058; doi:10.1186/s12968-021-00832-2)
Supplement: Supplementary file 4 — Additional file 4: Table S2. Correlation between 4D flow parameters and PV annulus diameter, MPA stiffness based on PWV index and pulmonary regurgitation parameters in repaired tetralogy of Fallot (rTOF). [file 12968_2021_832_MOESM4_ESM.docx]

**Table S2.** Correlation between 4D flow parameters and PV annulus diameter, MPA stiffness based on PWV index and pulmonary regurgitation parameters in repaired tetralogy of Fallot (rTOF).

| **4D flow parameters** | **Correlation with PV annulus diameter, cm** | | **Correlation with MPA stiffness, m/s** | | **Correlation with PRF, %** | | **Correlation with PRV, ml** | |
| --- | --- | --- | --- | --- | --- | --- | --- | --- |
|  | ***R*** | ***P*** | ***R*** | ***P*** | ***R*** | ***P*** | ***R*** | ***P*** |
| RV direct flow, % | -0.072 | 0.578 | -0.176 | 0.184 | 0.116 | 0.364 | 0.035 | 0.785 |
| RV retained inflow, % | 0.154 | 0.232 | -0.076 | 0.567 | -0.078 | 0.544 | 0.004 | 0.974 |
| RV delayed ejection flow, % | -0.028 | 0.828 | -0.090 | 0.499 | 0.044 | 0732 | 0.009 | 0.945 |
| RV residual volume, % | 0.002 | 0.985 | -0.225 | 0.086 | -0.080 | 0.533 | -0.034 | 0.794 |
| RV peak systolic KEi_EDV_, µJ/ml | 0.103 | 0.428 | -0.041 | 0.759 | -0.045 | 0.726 | 0.034 | 0.789 |
| RV average systolic KEi_EDV_, µJ/ml | 0.049 | 0.706 | -0.044 | 0.743 | -0.086 | 0.501 | -0.043 | 0.741 |
| RV peak E-wave KEi_EDV_, µJ/ml | **0.358** | **0.004** | -0.033 | 0.805 | **0.465** | **<0.001** | **0.496** | **<0.001** |
| KE discordance | **-0.343** | **0.006** | -0.221 | 0.093 | 0.006 | 0.964 | -0.084 | 0.515 |

*KE* kinetic energy, *KE discordance* RV/LV systolic KEi_EDV_, *KEi_EDV_* kinetic energy normalized to end-diastolic volume (EDV), *LV* left ventricle, *MPA* main pulmonary artery, *PRF* pulmonary regurgitation fraction, *PRV* pulmonary regurgitation volume, *PV* pulmonary valve, *PWV* pulse wave velocity, *RV* right ventricular.
